# Face and content validity of a prospective multidimensional performance instrument for service delivery in district health systems in low-income countries: a Delphi study

**DOI:** 10.1093/inthealth/ihz064

**Published:** 2019-07-24

**Authors:** Elias Ali Yesuf, Eva Grill, Günter Fröschl, Damen Haile-Mariam, Daniela Koller

**Affiliations:** CIH^LMU^ Center for International Health, Ludwig-Maximilians-Universität München, Ziemssenstr. 1, München, Germany; Department of Health Policy and Management, Jimma University, Aba Jifar 1 Street, Jimma 378, Ethiopia; Institute for Medical Data Processing, Biometry and Epidemiology, Ludwig-Maximilians-Universität München, Marchioninistr. 17, München, Germany; CIH^LMU^ Center for International Health, Ludwig-Maximilians-Universität München, Ziemssenstr. 1, München, Germany; Division of Infectious Diseases and Tropical Medicine, Medical Centre of Ludwig-Maximilians-Universität München, Leopoldstr. 7, München, Germany; School of Public Health, Addis Ababa University, Zambia street, Addis Ababa, Ethiopia; Institute for Medical Data Processing, Biometry and Epidemiology, Ludwig-Maximilians-Universität München, Marchioninistr. 17, München, Germany

**Keywords:** Delphi study, district, health systems, indicators, low-income country, validity

## Abstract

**Background:**

Valid performance indicators help to track and improve health services. The aim of this study was to test the face and content validity of a set of performance indicators for service delivery in district health systems of low-income countries.

**Methods:**

A Delphi method with three stages was used. A panel of experts voted (yes vs no) on the face value of performance indicators. Agreement on the inclusion of indicators was a score of >75% and ≥50% during stages one and two, respectively. During stage three, indicators with a mean score of ≥3.8 on a five-point scale were included. The panel also rated the content validity of the overall set of indicators.

**Results:**

The panel agreed on the face value of 59 out of 238 performance indicators. Agreement on the content validity of the set of indicators reached 100%. Most of the retained indicators were related to the capacity of health facilities, the quality of maternal and child health services and HIV care and treatment.

**Conclusions:**

Policymakers in low-income countries could use a set of performance indicators with modest face and high content validity, and mainly aspects of capacity and quality to improve health service delivery in districts.

## Introduction

According to the health systems dynamics framework, health systems have 10 elements that interact in a dynamic way. These are: (1) goals and outcomes, (2) values and principles, (3) service delivery, (4) the population, (5) the context, (6) leadership and governance and (7–10) the organization of resources (finances, human resources, infrastructure and supplies, knowledge and information).^1^ At the core of a central axis of these elements is ‘organization and delivery of health care services’.^1^ Even although health systems are broadly defined, a healthcare system is assumed to be a subsystem within the health system that has fewer elements.

According to Wendt, Frisina and Rothgang,^2^ a healthcare system has elements of ‘financing, health service provision and regulation’. Similar to the health system, health service provision is the key element of a healthcare system. This concept is generally applied to national healthcare systems. Nevertheless, it can also be applied to subnational health systems such as district healthcare systems, which are at the forefront of primary health service delivery.^3^

‘A district health system includes the interrelated elements in the district that contribute to health in homes, educational institutions, workplaces, public places and communities, as well as in the physical and psychosocial environment.’^4^

The district health systems of low-income countries commonly have elements of service delivery, regulators, suppliers and financiers. In this study we focused on service delivery because it is the core function of district health systems. Service delivery can be executed by private or public providers. Public providers of health services are generally the major providers of health service in districts of low-income countries and commonly include health posts, health centres, district hospitals and district health offices, which are vertically integrated for planning and control.

The performance of a health system is the attainment of multiple goals and the distribution of the attainment of those multiple goals.^5^ It includes access to and capacity for the provision of quality healthcare as well as striving for better health status outcomes in an efficient and equitable manner.^6–8^

Performance measures are important for service delivery in district health systems to capture areas of success that could be scaled up, and also to learn from failures.^9^ They also help decision-makers to use the results of performance measurement for decision-making at the local level.^10^ Moreover, they can be applied to elements in the district health system to improve quality outputs, such as growth monitoring and institutional delivery by attaching the results of performance measurement to incentives for staff.^11^

District health systems in low-income countries face a myriad of challenges, including difficulty in integrating vertical programmes,^12^ ambiguity regarding their administrative roles^13^ and poor performance monitoring.

In Uganda, a perceived lack of local decision space contributed to poor performance monitoring in district health systems.^14^ Poor performance monitoring of health service delivery leads to weak accountability for results and a decline in the quality of services. In low- and middle-income countries (LMICs), the poor quality of health services contributed to 5 million annual deaths.^15^ Past efforts at developing performance indicators for low-income country health systems were made by organizations such as the WHO^16^ and individuals such as Kruk and Freedman.^17^ However, the indicators are not validated for health systems of low-income countries.

Other indicators tend to be process oriented. For example, performance indicators used at the national and subnational level in a low-income country such as Ethiopia are process oriented, for instance, the number of deliveries in health institutions.^18^

Therefore, there is a need for a set of valid indicators for district health systems in low-income countries with both process and outcome attributes.

The aim of this study is to test the face and content validity of a set of performance indicators for district health systems in low-income settings to help improve performance monitoring of service delivery towards the goal of better health and longevity.

## Methods

### Working definitions of performance dimensions of district health systems

The following are key terms and their definitions as used in this paper.

Service delivery has access, capacity and quality dimensions that partially lead to better outcomes in health status; these should be equitably distributed, and resources should be used efficiently to produce better health status.^19^

Access (to health services): ‘the perceptions and experiences of people as to their ease in reaching health services or health facilities in terms of location, time, and ease of approach’.^20^

Capacity refers to ‘skills, tools and processes’ that need to be in place in a functioning system.^21^

Quality: ‘[t]he correct provision of evidence-based healthcare services to all who could benefit, but not to those who would not benefit’.^6^

Outcomes: the incidence and prevalence of conditions and diseases and their risk factors as well as subjective health status and objective health status such as mortality.

Efficiency: the relationship between inputs and outputs of healthcare.^6^

Equity: ‘[t]he absence of systematic differences in one or more aspects of health status (or access) across socially, demographically or geographically defined population groups’.^20^

### Development of the prospective instrument

The dimensions of the performance of a health system can be measured by a set of indicators. For an indicator to be selected as a performance indicator the following criteria are commonly applied: importance, relevance, validity, reliability and feasibility.^22^ In 2016 and 2017, we used a narrative systematic review and qualitative interviews to identify relevant performance indicators for service delivery in district health systems of low-income countries. Those performance indicators that were identified were categorized into access, capacity, quality, outcomes, equity and efficiency dimensions of health system performance, and used as part of the prospective instrument for this study. The instrument was used as a background paper for voting and commenting on the Delphi method.

### Study design

A Delphi method was chosen to overcome geographical and logistical difficulties since experts from many different countries were involved. In this situation the Delphi method was preferable to face-to-face discussions.^23^

### Setting

This study was set up in the district health systems of low-income countries.

### Panel size and sampling of participants

Given that the target population of experts from which the panel was drawn was not known,^24^ panel size was not calculated a priori. Even though there was no recommended sampling technique for Delphi method studies,^25^ a panel of experts was selected using purposive sampling based on pre-set criteria. The criteria for an expert was somebody who (1) had published an article on the quality or performance of health systems in a low-income setting, (2) had experience working in a district health system (for example, as an administrator of a district health office) or (3) was recommended by either (1) or (2). Experts were recruited online via email. Experts acquired their experience in LMICs such as Afghanistan, Cameroon, Ethiopia, Ghana, India, Kenya, Nigeria, Pakistan, Rwanda, South Africa and Uganda.

### Variables

Face validity was defined as the ability of a performance measure to fall into the broader construct of service delivery performance in district health systems in low-income settings. Content validity was defined as the completeness of an entire set of performance measures to represent the service delivery performance of district health systems in low-income settings.

### Data collection and analysis

The email collector option of SurveyMonkey (One Curiosity Way, San Mateo, CA, USA) was used for data collection. Participants were provided with a background paper containing a set of relevant performance indicators of district health systems in low-income settings. They were asked to vote and comment on performance indicators over three stages.

During stage 1, participants were asked to vote (yes vs no) on each of the performance indicators regarding the ability of an indicator to fall under the broader construct of service delivery performance of district health systems in low-income countries and to rate the completeness of the entire set of performance indicators. Moreover, they were asked to comment on each of the performance indicators. Agreement on an indicator level was established when an indicator achieved >75% of the votes. An indicator polling 40–75% of the votes was considered equivocal and voted on again during stage 2. When an indicator only scored <40% of the votes it was considered to be excluded by the panel.

During stage 2, participants were given the set of performance indicators which took 40–75% of the votes during stage 1^26^ and they were voted on again. Moreover, performance indicators that had been proposed during stage 1 were voted upon, and those which scored <50% were excluded. Stage 2 panellists were also asked to comment on the performance indicators.

During stage 3, participants were asked to rate those performance indicators which received either >75% during stage 1 or ≥50% of the votes during stage 2. Furthermore, performance indicators that had been proposed during stage 2 were also rated. The panel was asked to rate the indicators on a five-point scale (from 1=least well to 5=very well), and comment on them. Performance indicators which achieved a mean score of ≥3.8 were retained for the final set of validated indicators.^27^

During each stage participants rated the prospective instrument on a four-point Likert scale (1=strongly disagree, 2=disagree, 3=agree and 4=strongly agree) to test its overall completeness.

The Delphi stages lasted an average of 5 weeks.

## Results

### Response rates and characteristics of the panellists

The overall median response rate was 9.8%. The ages of the panellists ranged from 33 to 58 y (mean=42.0, SD=9.8), 34 to 72 y (mean=51.6, SD=14.4) and 28 to 62 y (mean=39.2, SD=9.6) for stages 1, 2 and 3, respectively. Their work experience ranged from 10 to 35 y (mean=17.9, SD=9.3), 10 to 45 y (mean=27.4, SD=13.6) and 7 to 30 y (mean=15.3, SD=6.4) for stages 1, 2 and 3, respectively. Women represented 13, 30 and 14% of panellists during stages 1, 2 and 3, respectively. About 88, 50 and 78% of panellists were from Ethiopia during stages 1, 2 and 3, respectively. Workers in the government sector accounted for 50, 60 and 50% of panellists during stages 1, 2 and 3, respectively (Table 1).

**Table 1.** Characteristics of the panellists

Characteristics	Stage 1 (n=8) frequency (%)	Stage 2 (n=10) frequency (%)	Stage 3 frequency (%)
Gender			n=7
Male	7.0 (87.5)	7 (70.0)	6.0 (85.7)
Female	1.0 (12.5)	3 (30.0)	1.0 (14.3)
Country			n=9
Ethiopia	7 (87.5)	5 (50.0)	7.0 (77.8)
South Africa	1 (12.5)	2 (20.0)	0.0 (0.0)
USA	0 (0.0)	1 (10.0)	0.0 (0.0)
Canada	0 (0.0)	1 (10.0)	0.0 (0.0)
Belgium	0 (0.0)	1 (10.0)	1.0 (11.1)
Uganda	0 (0.0)	0 (0.0)	1.0 (11.1)
Sector			n=8
Government	4 (50.0)	6 (60.0)	4.0 (50.0)
Academia	2 (25.0)	3 (30.0)	2.0 (25.0)
Private for profit	0 (0.0)	0 (0.0)	1.0 (12.5)
Private non-profit	2 (25.0)	1 (10.0)	1.0 (12.5)

Response rates varied by stage and dimension of district health system performance. During stage 1, response rates ranged from 8 out of 95 (8.4%) for the equity dimension to 17 out of 95 (17.9%) for the capacity dimension; the median response rate was 9.5%. The stage 2 response rate ranged from 8 out of 95 (8.4%) for the health status outcomes dimension to 14 out of 95 (14.7%) for the capacity dimension; the median response rate was 10.5%. The stage 3 response rate was 9.3%.

### Findings of face validity

Panellists were provided with 238 indicators to vote upon during stage 1: 142 indicators (59.7%) were included, 88 (37.0%) were assigned to re-voting and 8 (3.4%) indicators were selected for exclusion. On the level of performance dimension, inclusion during stage 1 was highest for outcomes (79.6%) followed by equity (72.2%) and the lowest was for access (38.9%). In the re-voting category the highest was for capacity (53.1%) followed by access (50.0%) and the lowest was for outcomes (20.4%). See Table 2 for more information.

**Table 2.** Stage 1 indicators, inclusion and exclusion results

Performance domain	Number of indicators included (>75% of votes) frequency (%)	Number of indicators for re-vote (40–75% of votes) frequency (%)	Number of indicators excluded (<40% of votes) frequency (%)	Total
Capacity	14 (43.8)	17 (53.1)	1 (3.1)	32
Access	7 (38.9)	9 (50.0)	2 (11.1)	18
Quality	65 (56.5)	45 (39.1)	5 (4.4)	115
Outcomes	39 (79.6)	10 (20.4)	0 (0.0)	49
Efficiency	4 (66.7)	2 (33.3)	0 (0.0)	6
Equity	13 (72.2)	5 (27.8)	0 (0.0)	18
Total	142 (59.7)	88 (36.9)	8 (3.4)	238

Comments by panellists during stage 1 were integrated with the findings of the votes. For example, a participant during stage 1 suggested that ‘the indicators should include indicators for the role of other stakeholders like non-governmental organizations and the indicators should focus on [the] healthcare system not the health system’. However, regarding the role of other stakeholders, it was noted that an indicator concerning the existence of intersectoral coordinating bodies in the district health office already existed.

Stage 1 panellists also suggested including certain indicators, most of which were accepted, except when there was already an indicator in place. For example, an indicator called ‘the percentage of children who completed the pentavalent vaccine by the age of 1 y’ was included in stage 2. One panellist suggested removing indicators for vaccines which were not available in most low-income countries; for example, the measles–mumps–rubella vaccine was not available in district health systems in Ethiopia and thus was removed from the set of indicators. See Supplementary File 1 for the comments made and actions taken during stage 1.

During stage 2, 104 indicators were provided for panellists to vote upon, and it was agreed to include 99 (95.2%) of these. Performance dimension level agreement was 100% for access, outcomes, efficiency and equity dimensions of performance. Moreover, agreement was very high for capacity and quality dimensions (Table 3).

**Table 3.** Voting for indicators during stage 2

Performance domain	Number of indicators included (≥50% of votes) frequency (%)	Number of indicators excluded (<50% of votes) frequency (%)	Total
Capacity	20 (95.2)	1 (4.8)	21
Access	11 (100.0)	0 (0.0)	11
Quality	48 (92.3)	4 (7.7)	52
Outcomes	12 (100.0)	0 (0.0)	12
Efficiency	2 (100.0)	0 (0.0)	2
Equity	6 (100.0)	0 (0.0)	6
Total	99 (95.2)	5 (4.8)	104

During stage 2 the panel also commented on the indicators, and suggested including, excluding or merging indicators. For example, an indicator concerning the existence of a quality committee in the district health office to address quality problems was added, and indicators regarding children without health insurance and underinsured adults were merged with the one for families without health insurance. See Supplementary File 2 for a detailed description of the comments made and actions that were taken.

The stage 3 panel was provided with descriptions of the functions of district health offices and public providers (health centres, health posts and women community health volunteers) in district health systems in Ethiopia.

The panel voted for 241 indicators to be included during stages 1 and 2. Fourteen indicators were either merged or removed based on comments made during either stage 1 or stage 2. Therefore, stage 3 started with 227 indicators, which were organized by elements of public provider of district healthcare, and whole health system indicators on outcomes, efficiency and equity; 57 indicators (25.1%) with a weighted mean score of ≥3.8 were retained. Agreement regarding the percentage of indicators included for the level of each service provider in the district health system was consistent with agreement overall, each of which scored in the upper 20s, except for the efficiency and equity indicators for the whole system. Details of the indicators retained and excluded during stage 3 are provided in Table 4. There were no substantial comments made during stage 3 (Supplementary file 3).

**Table 4.** Results of ratings during stage 3

Element of district healthcare system	Number of indicators included (weighted mean score ≥3.8) frequency (%)	Number of indicators excluded (weighted mean score <3.8) frequency (%)	Total
District health office	10 (23.8)	32 (76.2)	42
Health centre	31 (26.5)	86 (73.5)	117
Health post	2 (28.6)	5 (71.4)	7
Women community health volunteers	2 (28.6)	5 (71.4)	7
Outcomes: the whole system	11 (25.6)	32 (74.4)	43
Efficiency: the whole system	0 (0.0)	3 (100.0)	3
Equity: the whole system	1 (12.5)	7 (87.5)	8
Total	57 (25.1)	170 (74.9)	227

Indicators retained at the end of stage 3 are listed in Table 5, ranked by a matrix of performance dimension and the element of each public service provider in the district health system. Outcome and equity indicators partially attributed to the entire district health system are described in Table 6.

**Table 5.** Final list of valid indicators by performance dimension and the element of public service providers of the district health system

District health office	Health centre	Health post
Capacity indicators: Making available essential drugs, such as for the treatment of malaria Percentage of health centres which received support, including training and supervision Percentage of pregnant women who reached the receiving health facility among pregnant women referred by women development teams Percentage of sick children who reached the receiving health facility among sick children referred by women development teams	Capacity indicators: Health centre-developed checklist to assess services quality Quality indicators: Percentage of non-pregnant women who needed family planning services and taking at least one method Percentage of pregnant women with at least one antenatal care check-up during the last pregnancy Percentage of pregnant women with ≥4 antenatal care check-ups during the last pregnancy Percentage of pregnant women counselled on danger signs of pregnancy during antenatal care of the last pregnancy Percentage of pregnant women screened for HIV during the last pregnancy Percentage of pregnant women screened for syphilis during the last pregnancy Percentage of pregnant women screened for gestational diabetes mellitus during the last pregnancy Percentage of pregnant women screened for hypertension during the last pregnancy Percentage of pregnant women tested for blood group and type during the last pregnancy Percentage of pregnant women screened for iron deficiency anaemia during the last pregnancy Percentage of pregnant women supplied with iron during the last pregnancy Percentage of pregnant women supplied with folic acid during the last pregnancy Percentage of pregnant women with HIV/AIDS who received treatment Percentage of women who gave birth in health centre during the most recent pregnancy Percentage of women enrolled in postnatal care services immediately after birth Percentage of children who initiated vaccination within 45 d after birth Percentage of children who completed polio vaccine by age 2 y Percentage of children who completed measles vaccine by age 2 y Percentage of children who completed pneumococcal conjugate vaccine by age 2 y Percentage of children who completed pentavalent vaccine – combination of diphtheria, pertussis, tetanus, haemophilus influenza type B and hepatitis B –by age 2 y Percentage of children aged 45 d to 2 y who underwent regular growth monitoring Percentage of children with diarrhoea who received treatment Percentage of children with respiratory tract infection who required treatment and received treatment Percentage of HIV-positive adults lost to follow-up after initial positive test and then located with tracking Percentage of HIV-positive adults receiving highly active antiretroviral therapy Percentage of HIV-positive adults screened for TB in the past year Percentage of HIV and TB coinfected adults who received anti-TB treatment Percentage of TB patients counselled on transmission of the disease to others Percentage of newly diagnosed TB on anti-TB treatment of directly observed therapy Percentage of patients on TB treatment who completed treatment	Capacity indicators: Percentage of pregnant women in a village registered by health post Percentage of households in a village that received education on bednet utilization
Access indicators: Percentage of households within 30 min walking distance from a primary healthcare provider Percentage of children with geographic access to vaccination services Health officer density per 1000 population Proportion of households covered by community health workers through outreach activities		Women community health volunteers, capacity indicators: Percentage of pregnant women in a village detected by community health volunteers Percentage of pregnant women referred to health post or health centre among pregnant women detected by community health volunteers
Quality indicators: Percentage of households in high-risk communities sprayed with indoor residential spraying Annual TB detection rate in the district		

**Table 6.** District healthcare system outcome and equity indicators

District healthcare system outcome indicators
Rate of consistent toilet utilization
Regular bednet utilization rate
Measles cases per 100 000 population
TB cases per 1000 population
Trachoma cases per 100 000 population
Onchocerciasis cases per 100 000 population
Perinatal mortality rate
Rate of stillbirths
Neonatal mortality rate
Percentage of deaths within 28 d of live births weighing <1500 g
Postneonatal mortality rate
Infant mortality rate
Maternal mortality ratio
District healthcare system equity indicator
Percentage of pregnant women who received antenatal care: urban vs rural

### Content validity

Six out of seven members of the panel agreed that the set of indicators in stage 1 reflected service delivery performance in district health systems. Seven out of eight agreed on the overall validity of the set of indicators during stage 2. During stage 3, all eight participants agreed that the final set of indicators reflected service delivery performance in district health systems in low-income countries.

## Discussion

Average face validity increased from stage 1 to stage 2 and then decreased from stage 2 to stage 3. At the dimension level, agreement during stage 1 was highest for the outcome dimension followed by equity, with suggestions made for new quality indicators. During stage 2, agreement at the dimension level increased to 100% for all of the dimensions except for capacity and quality. During stage 3, about a quarter of the indicators from each dimension received a high rating, except for efficiency and equity.

During each subsequent stage there was an increase in consensus on content validity, indicating that the entire set of indicators represented the broader construct of service delivery performance in district health systems in low-income settings.

Finally, 59 indicators were retained. All performance dimensions were addressed except efficiency. A little more than half of the indicators concerned health centres, reflecting the core function they play in districts regarding service delivery of primary healthcare. The indicators are comparable with international norms such as 100 core health indicators^28^ and WHO indicators for monitoring the six building blocks of health systems.^16^

This study found that access indicators commonly used by developing countries^17^, such as 1 physician per 1000 people of the population, were found to be valid; these are also suggested by WHO for monitoring the health workforce.^16^ WHO also suggest monitoring services such as antenatal care, delivery, HIV/AIDS and TB treatment, which were also found to be valid in this study. Outcome indicators, such as infant mortality and maternal mortality, which were found to be valid for low-income settings in this study, are also commonly used in developing countries.^17^

The panel selected several indicators related to the quality of healthcare. This is important with regard to health centres because they are the major providers of healthcare in districts and therefore should be monitored. Indicators related to antenatal care and child health were also selected. This is important because of high maternal and child health mortality in low-income countries.

Even although district healthcare systems in low-income countries are affected by efficiency and equity problems, no efficiency indicator and most of the equity indicators did not make it into the final set of indicators.

Implementation of the indicators by districts may help to strengthen the capacity of district health systems and improve health service quality and utilization. Together with subsidies, the indicators can be used to improve utilization rates for healthcare services across districts. For example, quality indicators such as growth monitoring and institutional deliveries, which were found to be valid in this study, were used in Rwanda to increase the use of those services by subsidising health centres to provide better performance.^11^ Moreover, quality indicators such as the percentage of TB patients who completed treatment could be a proxy for other aspects of the district health system, for example the capacity of the system to retain patients in treatment.^19^

Data availability might be a challenge in utilizing the indicators as most of the data collected in districts of low-income countries focus upon service delivery for mothers and children.^29^ However, application of the indicators in the face of a shortage of data at the district level still helps, in two ways. First, it pressurizes districts to explore new ways of using locally generated data. Second, it encourages them to identify areas in which data are lacking, particularly regarding outcomes. These encourage district health systems towards better monitoring of service delivery performance by linking it to healthcare outcomes such as mortality.

The findings of this study are limited by the fact that agreement on face validity did not show a monotonic increase. Moreover, the response rate in this study was less than response rates that have been reported in Delphi studies (of around 30%).^24^ This may have affected the inclusion or exclusion of some indicators through the effects of non-response bias. Hence, despite experts being invited from many LMICs, respondents were mostly from Ethiopia, South Africa and Uganda. Thus the indicators selected as being valid by the participants might have been more applicable to health systems in low-income countries in eastern and southern Africa. Moreover, service delivery in district health systems is only partially responsible for morbidity and mortality outcomes. Therefore, outcome indicators are only partly attributed to service delivery in district health systems. Finally, due to the small sample size, the inability to aggregate the selected indicators by the gender of the panel, i.e. to estimate the effect of gender upon the selection of a group of indicators, may also have limited this study’s findings.

### Conclusions

Policymakers in low-income countries could use a set of performance indicators with modest face and high content validity, and mainly aspects of capacity and quality, to improve health service delivery in districts. Policymakers in national and local settings should pilot the indicators and document the challenges, including the availability of data at the local level, and work on mechanisms to secure additional data, particularly regarding outcomes. Outcome indicators found to be valid in this study should be used with caution by applying the proportion of outcomes attributed to the district health system and other social sectors, such as education and economic status. This can be achieved by using panel regression techniques to determine the adjusted coefficient of health services in the health production function by using infant mortality as a dependent variable, and health services, economic status and levels of education as predictors.

## Supplementary Material

ihz064_District-Indicators-EAY-Supplementary_files-20190315
