# Supplementary material for: Face and content validity of a prospective multidimensional performance instrument for service delivery in district health systems in low-income countries: a Delphi study
Source: Int Health. 2019 Jul 24;12(3):184–91. doi: 10.1093/inthealth/ihz064 (PMC11973424; doi:10.1093/inthealth/ihz064)
Supplement: ihz064_District-Indicators-EAY-Supplementary_files-20190315 [file inthealth_12_3_184_s1.docx]

**Supplementary files**

**Supplementary file 1: Comments during stage 1 and actions taken**

| **S. No.** | **Comments** | **Actions** |
| --- | --- | --- |
|  | **Capacity dimension** |  |
| 1 | There is a capacity building given by different stakeholder and Non-Governmental Organizations but there is a gap. | Not accepted as the study focused on public service delivery in district health system |
| 2 | I haven't seen indicators on the role of other stakeholders like partners and the role of community and other sectors like education, agriculture etc. | There was an indicator for the role of community called existence of community level health committees. Moreover, cost-sharing by the community and resources of the community for health action had indicators.  Whether the set of indicators should include the role of other sectors was put forth for voting in stage 2 |
| 3 | I suggest the district healthcare system indicators be specific to what must happen in the healthcare system not in the health system. | Health status outcomes were attributed only partially to service delivery in district health systems in the third stage |
| 4 | Some of the indicators are critical and some are 'nice to have'. The challenge is in having a set of indicators that is not too large but appropriate and this will tend to vary according to the country in question. | We recommended to test the feasibility of the indicators for each low-income country |
| 5 | I think you are measuring the wrong things. I do not think infrastructure or having trainings or even specific activities are the way to assess capacity of district health offices. It is more about the quality of what they do and the way they support quality improvement at health facilities. Having specific things like trainings may be necessary but are not in my view appropriate measures of whether the system is working. | Questions were added to stage 2 asking the panel to suggest indicators on quality for district health offices and health centers. |
| 6 | It's important if possible we may add an indicator of hygiene and sanitation program like latrine utilization. | Rate of consistent toilet utilization already included in outcome dimension |
| 7 | Child indicators are missing, i.e. percentage of cases of diarrhea treated. | There was already an indicator on percentage of children who needed treatment for diarrhea and received treatment under quality dimension |
| 8 | Measures such as Percentage of pregnant women referred are inadequate. What you are interested in is whether women who are referred which actually go to the site to which they are referred so the measure is referrals completed. | Percentage of pregnant women who reached receiving health facility among referred was included in stage 2 for voting |
|  | **Access dimension** |  |
| 9 | It is better if your indicators can address the dimension of access based on Donabedian major components of quality. | Donabedian was considered in the design of the performance definition as capacity and access were inputs, quality was a process, and outcomes were results |
| 10 | What about access to vaccination and proper health education on priority health issues? | Indicators on percentage of children with geographic access to vaccination services and percentage of the population with access to health education on priority health issues were included during stage 2 for voting |
| 11 | Percentage of households (with adults) within 30 minutes of walking distance from a primary healthcare provider (either health post, or health center, or district hospital) this indicator should apply for the lowest level of health facility (health post) not for hospital. | This indicator was voted to be included in stage 1. Therefore, it is modified and included in stage 3 as percentage of households within 30 minutes of walking distance from a primary healthcare provider –either health center or health post |
| 12 | The distance/time taken should be different for different levels. | Not accepted because it was difficult to define distance and time for each level of the service providers in the health system |
| 13 | These indicators are very site specific. What about a country that does not have insurance but a good system of care? | The indicator was modified as proportion of the population with public or private health insurance, if available to reflect the context |
|  | **Quality dimension** |  |
| 14 | I find myself choosing No because many of these indicators have denominators that cannot be known by the District Health Services, or concern services that may not be provided. This is very context specific. | Denominators would be known from census and population projections |
| 15 | It is better if children complete vaccination at the age 1. | Percentage of children who completed by age one of pentavalent vaccine included in stage 2 for voting |
| 16 | Better to add required life skills indicators for adolescents to lead health life and transit to adulthood also good to consider accident related indicators as adolescents are at risk of accidents due to their age factor. | Availability of interventions to promote adolescent competence, confidence, connection, character and caring in the district healthcare system, percentage of adolescents who sustained road injury, percentage of adolescents who drowned, percentage of adolescents involved in at least one interpersonal violence were included in stage 2 for voting |
| 17 | Indicators numbered 4110 [completion of Measles-mumps-rubella vaccine] and 4114 [completion of Varicella vaccine] seem to have a concern of feasibility as the antigens may not be provided in the Ethiopian context. Further, there seem to be no indicator for Pentavalent vaccine. Indicator 4120 [Percentage of children treated for malnutrition using ready-to-use therapeutic food] may need revision to reflect the denominator "among malnourished children." | Vaccines Measles-mumps-rubella and Varicella were not available in district healthcare systems in Ethiopia. These indicators were removed.  Indicator for pentavalent vaccine was included for voting in stage 2.  Denominator was added to the indicator percentage of children treated for malnutrition using ready-to-use therapeutic food |
| 18 | Percentage of HIV positive children who receive treatment add indicators which track TB in children and adolescents. | There was already an indicator on HIV in children. And there was also an outcome indicator on percentage of the population (both children and adults) with tuberculosis |
| 19 | Indicators related to maternal health like cervical cancer but I didn't have an information. | There was already indicator on screening of cervical cancer among women |
| 20 | How about empowerment of women in decision making at least on health seeking? | Percentage of women who received empowerment message on health seeking from a health facility added to stage 2 for voting |
| 21 | Percentage of HIV treated women. | Percentage of women with HIV/AIDS who received treatment added to stage 2 for voting |
| 22 | Issue of being treated in a respectful and compassionate manner is not seen | Percentage of patients who reported that doctor-always listened, explained, showed respect, and spent enough time already existed |
| 23 | At PHC level in districts patients are mostly treated by nurse clinicians, as doctors are too few. Need more nurse related indicators | Panel was asked to suggest nurse related indicators during stage 2 |
|  | Outcomes dimension |  |
| 24 | It is better if you consider potential life years lost due to accident related disability and death as indicator. | Potential years of life lost due to accident related disability and potential years of life lost due to accident related death were added to stage 2 for voting |
|  | Equity dimension |  |
| 25 | Better if you can consider Health facility with all whether road access both in rural and urban. | Percentage of households with geographic access to health center which has all weather road (urban vs. rural) was added to stage 2 for voting |
| 26 | Equity measures reflect the impact of social determinants of health and hence are best used to measure the performance of the general health system (including the effect of interventions in other sectors) rather than the more focused healthcare system | The study was focused on service delivery district health system though the health status outcomes were partially attributed to the health system. Thus the comment was not accepted. |
|  | **General comments** |  |
| 27 | If possible add an indicator of hygiene and sanitation program and health insurance activity | Hygiene indicators like consistent hand washing and standard toilet utilization were already included in outcomes dimension. Moreover, insurance related indicator like percentage of households with health insurance existed in access dimension |
| 28 | Proportion of healthcare workers satisfied with their job, proportion of healthcare workers who intend to leave their job in the coming 12 month  are necessary. | Percentage of healthcare workers satisfied with their job, and percentage of healthcare workers who intend to leave their job in the coming 12 months were included to stage 2 for voting |
| 29 | As commented under some of the specific themes the indicators need to be checked whether they qualify the criteria for a good indicator such as specificity, feasibility, sensitivity, comparability, etc... | The objective of this study was on validity. Therefore, issues of feasibility and comparability will be addressed in future studies |
| 30 | Basically, the problem with the survey is that it mixes the responsibilities of the district health authorities with the responsibilities of the health facilities. | We have organized the indicators by level of public service providers in district health system in stage 3 |

**Supplementary file 2: Comments and actions taken during stage 2**

| **S. No.** | **Comments** | **Actions taken** |
| --- | --- | --- |
|  | **Capacity** |  |
| 1 | Do you have quality committees to measure quality problems and design ways to address them? | Indicator called existence of quality committees in district health office to measure quality problems and design ways to address them was included in stage 3 for rating |
| 2 | Number of health facilities developed checklist to assess services quality | Health center developed checklist to assess services quality was added to stage 3 for rating |
| 3 | Why not include indicators relevant to child referral | Percentage of sick children in a village detected by community health volunteers,  Percentage of sick children in a village referred to health post or health center among sick children detected by community health volunteers, and  Percentage of sick children who reached receiving health facility –health post or health center- among sick children referred by community health volunteers were added to stage 3 for rating |
|  | **Access** |  |
| 4 | Most of the indicators about the availability and use of health insurance is currently a wishful thinking in Ethiopia. The indicators you mentioned serves better if they were to be used in a country with near universal health insurance coverage and with the services to go with it. | Indicators on percentage of children without health insurance, percentage of adults insured all year, and percentage of adults underinsured were removed and included in percentage of families with health insurance (if available) and percentage of families underinsured |
|  | **Quality** |  |
| 5 | I said no to two indicators since these are not priorities in the setting you are targeting | Indicator on uterine cancer screening was removed as it did not go with low-income country health policy priority |
|  | **Outcomes** |  |
| 6 | These are important indicators but very expensive surveys are needed for reliable information at district level. Unless you have resources to do that, none of these questions should be used as indicators | Indicators on Potential Years of Life Lost due to accident related disability and Healthy Life Expectancy at Birth were removed because they need expensive surveys and calculations for which districts may not have resources. |
|  | Efficiency |  |
| 7 | Question 62 is confusing. An appropriate one will be average hospital days weighted by severity of conditions. | Number of hospital days divided by number of discharges weighted by severity was removed because most health centers do admissions rarely, and district hospitals are found in a few districts and they are directly controlled by regional health offices which are not in the authority of district health offices |
|  | **General** |  |
| 8 | Too many indicators for any survey and need to consider resources needed to get the indicators and test their appropriateness in each context. | Indicators on completion rate of Diphtheria-pertussis-tetanus, Hemophilus influenza type B, and Hepatitis B were removed and Percentage of children who completed by age 2 of pentavalent vaccine was kept because pentavalent vaccine is a combination of the vaccines stated above. |
|  |  | Moreover, indicator called “collaborate with universities and other learning institutions on training related with Maternal and Child Health” was implied in another indicator called “organize trainings, conferences and workshops on current healthcare issues for health professionals (health officers, nurses, and midwives).” Therefore, the first was removed and the latter was kept |

**Supplementary file 3: Stage 3 comments and actions**

| **S. No** | **Comments** | **Actions taken** |
| --- | --- | --- |
|  | **District health office** |  |
| 1 | Some of the indicators are composite and also few indicator looks subjective | Nonspecific comment. No action taken. |
| 2 | Some of the indicators, especially perceived health spending by the poor compared to the rich looks poor measurement as the definition of poverty is not well defined or at least defined in Ethiopia | This indicator was rated to be excluded by the participants |
| 3 | The indicators are relevant for developing countries , they measure the prevailing health system | No action taken |
|  | **Health center** |  |
| 4 | Many of the indicators that I have valued as least well indicators appeared to me very difficult to collect data for measurement. The health information system must be advanced enough to provide such data. otherwise the cost outweighs the benefit of measuring them | Feasibility was not the objective of this study. In future study we will address the feasibility issue. |
|  | **Health post** |  |
| 5 | It's better to add Neglected tropical disease (NTD) like trachoma Onchocerciasis | Rate of trachoma and rate of onchocerciasis were added to the final set of indicators |
| 6 | I would say the indicators for health posts are too few. You have not touched upon each of the 16 packages. For example number of households with latrine, number of model families in the kebele etc are critical | We already had the rate of consistent toilet utilization under outcomes |
|  | **Women community health volunteers** |  |
| 7 | It's better to add environmental sanitation indicator and ITN utilization | We already had regular bed net utilization rate as an indicator under outcomes |
|  | **Outcomes** |  |
| 8 | It's good but in some indicator were unknown at district level like smoking rate alcohol consumption | Both indicators were rated to be excluded during this stage |
|  | **Equity** |  |
| 9 | These measurement indicators cannot be calculated using routine health service data. all need a survey or a special study which is costly | Equity indicators were rated to be excluded during this stage except one which was percentage of pregnant women who received antenatal care: Urban vs. rural. This indicator can be drawn from routine data |
|  | All the indicators are crucial to measurement of equity of the health system | Partially taken as all except one equity indicator were rated to be excluded |
|  | **General** |  |
| 10 | I felt stalked about filling this questionnaire | Not taken because it is non-specific |
